# Comparative safety of multiple doses of erythropoietin for the treatment of traumatic brain injury: A systematic review and network meta-analysis

**DOI:** 10.3389/fneur.2022.998320

**Published:** 2022-12-13

**Authors:** Qingyong Zheng, Dan Duan, Jianguo Xu, Xing Wang, Yonggui Ge, Lu Xiong, Jingjing Yang, Saimire Wulayin, Xiaofeng Luo

**Affiliations:** ^1^School of Public Health, Lanzhou University, Lanzhou, Gansu, China; ^2^Evidence-Based Nursing Center, School of Nursing, Lanzhou University, Lanzhou, Gansu, China; ^3^Evidence-Based Medicine Center, School of Basic Medical Sciences, Lanzhou University, Lanzhou, Gansu, China; ^4^The First Clinical Medical College of Lanzhou University, Lanzhou, Gansu, China; ^5^Department of Rehabilitation, The First Hospital of Lanzhou University, Lanzhou, Gansu, China; ^6^The Second Clinical Medical College of Lanzhou University, Lanzhou, Gansu, China

**Keywords:** erythropoietin, traumatic brain injury, optimal dose, safety, systematic review, network meta-analysis

## Abstract

**Introduction:**

Over the past few decades, advances in traumatic brain injury (TBI) pathology research have dynamically enriched our knowledge. Therefore, we aimed to systematically elucidate the safety and efficacy of erythropoietin (EPO) dosing regimens in patients with TBI.

**Methods:**

Data search included PubMed, the Cochrane Library, Embase, Web of Science, and ClinicalTrials.gov for related research published before July 2022. The network meta-analysis was conducted using ADDIS 1.16.8, and the CINeMA tool was used to assess the quality level of evidence.

**Results:**

A total of six RCTs involving 981 patients were included in the network meta-analysis. EPO did not significantly reduce mortality in patients with TBI, but its risk of death decreased with increasing dosage (odds ratio (OR) of 12,000u vs. placebo = 0.98, 95% CI: 0.03–40.34; OR of group 30,000u vs. placebo = 0.56, 95% CI: 0.06–5.88; OR of 40,000u vs. placebo = 0.35, 95% CI: 0.01–9.43; OR of 70,000u vs. placebo = 0.29, 95% CI: 0.01–9.26; OR of group 80,000u vs. placebo = 0.22, 95% CI: 0.00–7.45). A total of three studies involving 739 patients showed that EPO did not increase the incidence of deep vein thrombosis in patients with TBI. However, the risk tended to rise as the dosage increased. Another two studies demonstrated that EPO did not increase the incidence of pulmonary embolism. The quality of evidence for all outcomes was low to moderate.

**Conclusion:**

Although the efficacy of EPO was not statistically demonstrated, we found a trend toward an association between EPO dosage and reduced mortality and increased embolic events in patients with TBI. More high-quality original studies should be conducted to obtain strong evidence on the optimal dosage of EPO.

**Systematic review registration:**

https://www.crd.york.ac.uk/PROSPERO/display_record.php?RecordID=272500. The study protocol was registered with PROSPERO (CRD42021272500).

## Introduction

Traumatic brain injury (TBI) refers to a series of blunt or sharp mechanical forces that induce vascular injury and hypoxia, leading to glial activation, primarily astrogliosis, inflammation, cell death, and tissue loss (1). It is worth noting that TBI may further cause cerebral herniation, cerebral edema, decreased cerebral perfusion pressure, and increased intracranial pressure in patients (2). TBI continues to plague millions of people worldwide and disproportionately affects the young, middle-aged, and elderly (3). Data show that about half of the global population experiences TBI once in their lifetime, resulting in substantial annual economic losses (4). TBI has been a pressing medical and public health problem globally and a leading cause of death and long-term disability (5).

Over the past few decades, advances in TBI pathology research have dynamically enriched our knowledge (6). TBI causes nearly irreversible brain damage, and its treatment and management remain significant challenges to clinicians. Therefore, several neuroprotective drugs have been extensively studied, focusing on early intervention in patients to protect brain nerve cells from secondary damage caused by ischemia and hypoxia (7). Erythropoietin (EPO) is a hemopoietin growth factor in the type 1 cytokine superfamily. It exists in the spleen, liver, bone marrow, and lung and is also expressed, to a lesser extent, in the brain, where it can elicit neuroprotective effects (8). Several randomized controlled trials (RCTs) (8–14) and meta-analyses (15–18) have found EPO to be efficacious in the treatment of patients with TBI. The findings were broadly similar, with EPO reducing mid-term mortality in patients with TBI. However, it did not significantly improve neurological function or increase the incidence of adverse events. However, the doses of EPO for TBI varied widely among the available studies. No studies have assessed the dose–response relationships in patients with TBI treated with EPO, and conventional meta-analyses have failed to elucidate the variation level in the efficacy of different EPO doses.

Network meta-analysis (NMA) enables a coherent ranking of multiple interventions, thus assisting decision-makers who wish to choose among various treatment options (14). Therefore, we performed an NMA to assess the efficacy of EPO in patients with TBI. It allows simultaneous comparison of the efficacy relationship between different doses to assess the effectiveness of EPO dosing regimens in treating patients with TBI.

## Materials and methods

This study was conducted according to the Preferred Reporting Items for Systematic Reviews and Meta-Analyses guidelines (PRISMA) (19). Ethical approval was not required since this review did not relate to individual patient data. This network meta-analysis was previously registered on the PROSPERO platform (**CRD42021272500**).

### Search strategy

We searched PubMed, the Cochrane Library, Embase, Web of Science, and ClinicalTrials.gov for RCTs using EPO for patients with TBI. The initial search was completed on 7 December 2020, and the search results were updated to 10 July 2022, using the automatic push function of the database weekly. Syntax and vocabulary were adjusted across databases, namely, “erythropoietin”, “Epoetin Alfa”, “Darbepoetin alfa”, “EPO”, traumatic brain injur^*^, “brain concussion”, “brain contusion”, “chronic traumatic encephalopathy”, “craniocerebral trauma”, penetrating head injur^*^, “basilar skull fracture”, “cerebrovascular trauma”, “traumatic intracranial hemorrhage”, and “TBI”, which were used individually or conjunctively. Supplementary Table 1 lists the search strategies used in this review. Related articles from the reference lists were also included to search for additional articles that the previously prespecified search strategy may not have retrieved.

### Study eligibility criteria

Studies that met the following criteria were included in our network meta-analysis: (1) patients with TBI requiring hospitalization, (2) RCTs compared the efficacy of EPO with placebo in patients with TBI, (3) studies should accurately report drug doses or calculate approximate doses based on the design regimen description, (4) significant clinical outcomes and adverse events were reported in the outcome indicators, and (5) all included studies should be written in English.

We excluded the following studies: (1) non-randomized trials, retrospective studies, case–control studies, review articles, case reports, and letters to the editor; (2) studies that did not explicitly report drug administration and dosing; and (3) studies involving patients with other concomitant serious diseases or at risk of initial thrombosis.

### Study selection

The initial search records were imported into EndNote X9 literature management software. In this study, two authors (QZ and DD) reviewed the titles and abstracts of the articles according to the inclusion criteria. Subsequently, full-text versions of all potentially relevant trials were obtained and examined to ensure that the study qualified for the network meta-analysis. We then selected the most recent data for analysis for the same trial reports with different follow-up periods. Any divergence regarding eligibility was resolved by discussion with a third reviewer (FX).

### Data extraction

We created a standard data table to collect related data including eligible studies characteristics (e.g., name of the first author, published year, type of research design, and follow-up time), characteristics of study participants (e.g., mean age, gender, and countries), drug administration (e.g., route, dose arrangement, total drug dose, and time to intervention), and reported clinical outcomes (e.g., adverse events). Any differences in the evaluation of these data were discussed until consensuses were reached.

### Quality evaluation

The risk of bias for each study was independently assessed by reviewers employing the identical bias risk assessment tool used for randomized trials from the Cochrane Handbook (RoB2) (20, 21). Risk of bias graphs showing the bias levels as low risk, high risk, and unclear risk, was generated using Review Manager 5.4 (Oxford, UK; The Cochrane Collaboration). Confidence In Network Meta-Analysis (CINeMA) version 1.9.1 (https://cinema.ispm.unibe.ch/) calculates the NMA contribution matrix by using the netmeta package of R software, based on the grading of recommendations, assessment, development and evaluation (GRADE) methodology for assessing the quality of evidence reported in the results (22). A comparison-adjusted funnel plot with the Egger test was constructed to assess for publication bias (23).

### The geometry of the network

We created evidence networks between comparisons using STATA (Stata Corp, College Station, Texas, USA, version 16.0) (24). A network plot was drawn presenting the interrelationships of comparisons across trials, which showed how each intervention was linked to the others through direct or indirect comparisons. In the network, each regimen is represented by a unique node, which means different nodes were designated for different dosages of EPO. Lines indicate direct head-to-head comparison of regimens, and the thickness of the line corresponds to the number of trials in the comparison. The node size corresponds to the number of samples involved in the intervention.

### Data synthesis and statistical analysis

ADDIS (IMI GetReal Initiative, EU, version 1.16.8) was used to calculate the safety by comparing different doses of EPO with placebo. ADDIS is rooted in the Bayesian theory and based on a model of the Markov chain Monte Carlo (MCMC) simulation method to obtain the corresponding combined effect sizes. The computational model adopted a random effect, rather than a fixed effect, model since it is the most conservative and appropriate analysis to account for variance among studies. The parameter setting of ADDIS is as follows: number of chains, 4; tuning iterations, 20,000; simulation iterations, 50,000; thinning interval, 10; inference samples, 10,000; and variance scaling factor, 2.5. The consistency model was used to pool data regarding mortality, pulmonary embolism, and deep venous thrombosis (DVT). Convergence was assessed using the potential scale reduction factor (PSRF), with a PSRF closer to 1 indicating better convergence and a PSRF < 1.2 denoting acceptable (25).

To improve the estimation accuracy of comparing relative effect sizes, we also created rank probabilities to derive recommendations for each outcome (25). These rankings included all treatments and were ordered according to their probability of being the best to the least. Consistency analysis was used to assess the overall consistency degree of the included studies, while node split analysis was used to estimate the robustness of the model (if closed loops exist in the network relationships), revealing possible differences among direct and indirect comparisons of specific nodes and their branches in the network, with a *p*-value of < 0.05 revealing inconsistencies in the network, which require further study (26).

## Results

### Study characteristics

**Figure 1:**
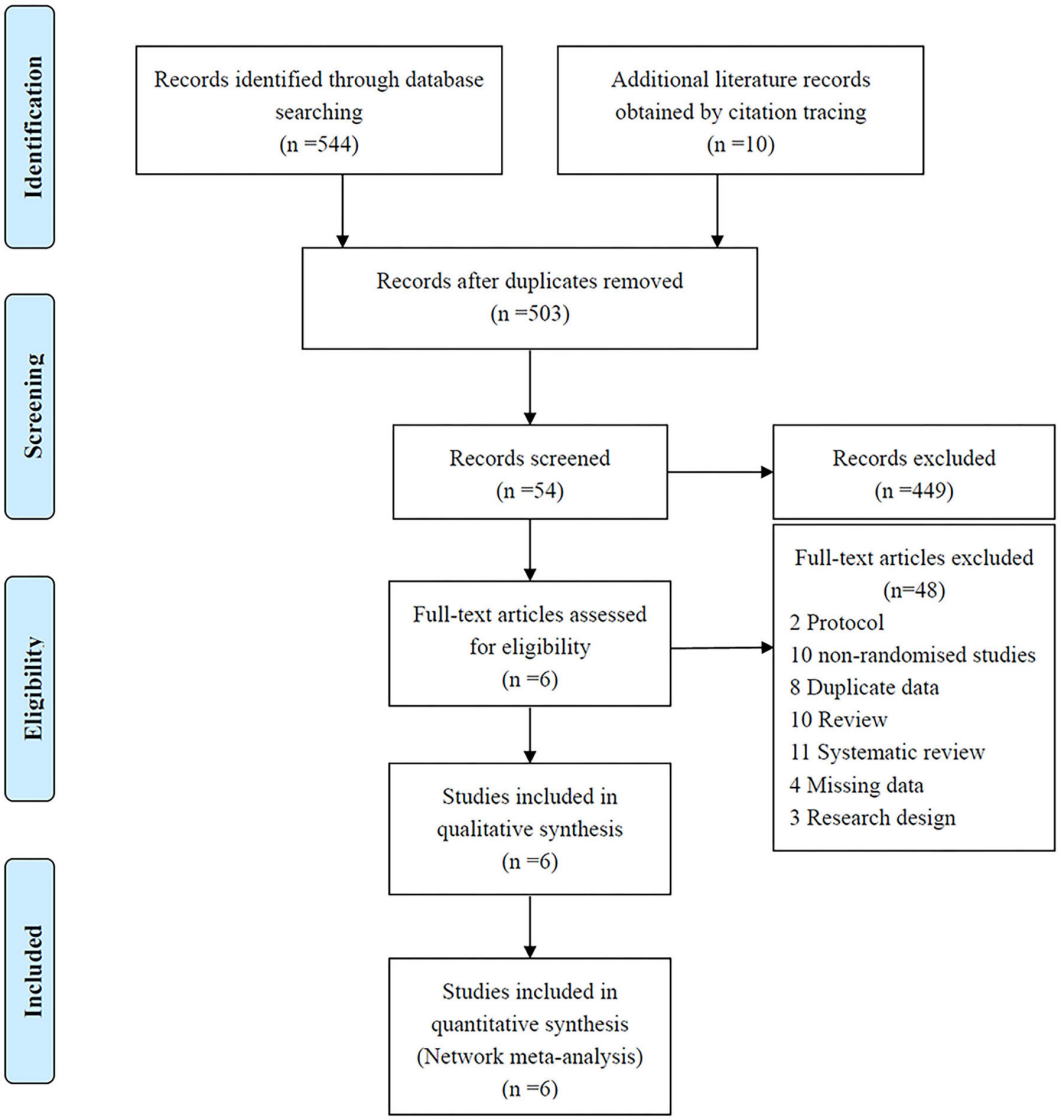
Flowchart of literature identification, review, and selection.

As shown in Figure 1, 544 studies were identified through the initial search, and 10 additional records were identified through citation tracking. After screening, we removed 51 duplicate records and excluded 449 studies that deviated from the inclusion criteria by reviewing the titles and abstracts. We reviewed the full text of the remaining 54 studies, 48 of which were removed due to non-randomized controlled trial designs or the unavailability of specific data. Finally, six RCTs (7, 9–12, 27) involving 981 patients were included. Table 1 summarizes the basic characteristics of the included studies.

**Table 1:** Baseline characteristics of included studies.

**References**	**Study design**	**Follow up**	**Inclusion criteria**	**Number of participants (EPO/Placebo)**	**Age (years)**		**Sex (males%)**		**Drug administration**				**Outcomes**
					**EPO**	**Placebo**	**EPO**	**Placebo**	**Route**	**Dosing regimen**	**Total dose**	**Time to intervention**	
Nirula et al. (11)	Double blind RCT single center	Discharge or dead	Moderate or sTBI: GCS < 13	16 (11/5)	35 ± 19	40 ± 26	8 (72.7)	3 (60)	IV	40,000u	40,000u	Within 6 h	1.Serum NSE, S-100B 2.ICP values 3.Adverse events 4.Mortality 5.ICU stay
Abrishamkar et al. (9)	Double blind RCT single center	Discharge or dead	sTBI with DAI: GCS (4–8)	54 (27/27)	25.2 ± 5.4	27.3 ± 4.0	-	-	SC	2,000u-Day 2, 4, 6, 8, 10 for 6 doses in 2 weeks	12,000u	About 5 h	1.RBC count, hematocrit, 2.thrombocyte count 3.GOS_‵_GCS 4.Adverse events: none 5.Mortality
Aloizos et al. (10)	RCT Multicenter	6 months	sTBI: GCS < 9	42 (24/18)	29.4 ± 1.3	46.5 ± 4.5	23 (95.8)	16 (88.8)	-	10,000u of EPO for 7 consecutive days	70,000u	-	1.GOS_‵_GOS-E 2.Adverse events: none 3.Mortality at 14 days 4.APACHE II score 5.ICP increasing 6.ICU stay
Nichol et al. (27)	Double blind, RCT Multicenter	6 months	Moderate or sTBI: GCS:3–12	603 (305/298)	30.5 (22.9-47.5)^*^	30 (22.9-48.3)	256 (83.9)	245 (82.5)	SC	40,000u Day 0 then weekly for max 3 doses	40,000u−147 80,000u−82 120,000u−75	Within 24 h	1.GOS-E 2.Adverse events 3.Mortality at 6 months
Li et al. (12)	Double blind RCT single center	3 months	sTBI: GCS ≤ 7	146 (75/71)	43.4 ± 10.1	41.1 ± 9.6	49 (65.3)	41 (57.7)	SC	EPO (100 u/kg) (average 6,000 units) on day 1, 3, 6, 9 and 12	30,000u	Within 2 h	1.Serum NSE, S-100B protein 2.Itemized GOS 3.mortality at 10 days 4.BP, hemoglobin level 5.Adverse events
Bai and Gao, (7)	Triple blind RCT single center	10 weeks	sTBI: GCS < 8	120 (60/60)	44.5 ± 11.4	43.1 ± 10.9	41 (68.3)	44 (73.3)	SC	6,000u, within 2 h, on days 3, 5,10, and 15	30,000u	Within 2 h	1.GOS score 2.Adverse events 3.Mortality at 10 weeks

RCT, randomized controlled trial; sTBI, severe traumatic brain injury; GCS, Glasgow Coma Scale; DAI, Diffuse axonal injury; NSE, neuron-specific enolase; ICP, intracranial pressure; ICU, intensive care unit; RBC, red blood cell; GOS, Glasgow Outcome Scale; GOS-E, Glasgow Outcome Scale Extended; APACHE, Acute Physiology and Chronic Health Evaluation; BP, blood pressure; IV, intravenous injection; SC, subcutaneous injection; u, units.

* Age range.

These included studies were published between 2010 and 2018, and the participants were distributed in the United States, Iran, Athens, Australia, New Zealand, France, Germany, Finland, Ireland, Saudi Arabia, and China. There were no significant differences in demographic characteristics, including age, sex, the severity of disease, and the Glasgow Coma Scale (GCS). The total EPO dosage among all the trials ranged from 12,000u to 80,000u. Medical treatment included intravenous and subcutaneous injections and was not reported in only one research (10). The total duration of drug interventions ranged from 1 to 7 sessions.

### Risk of bias and quality of evidence evaluation

**Figure 2:**
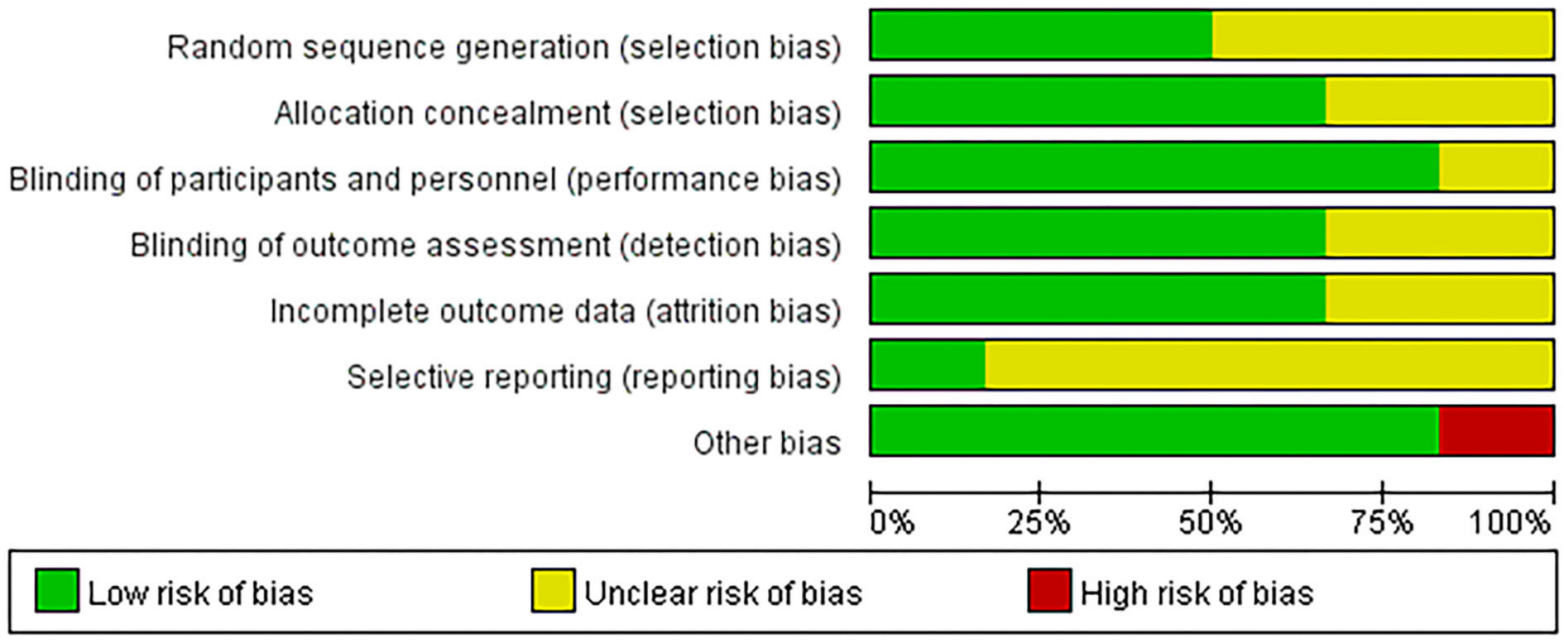
Risk of bias assessment results of included studies.

The evaluation results generally indicated an acceptable risk of bias among these RCTs (Figure 2). Of the six RCTs, three trials (7, 12, 27) (50%) were found to have a low risk of bias for randomization methods, four (7, 11, 12, 27) (66.7%) for concealment allocation, five (7, 9, 11, 12, 27) (83.3%) for blinding of participants and personnel, four (7, 9, 11, 27) (66.7%) for blinding of outcome assessment, four (9–11, 27) (66.7%) for incomplete outcome data, one (27) (16.7%) for selective reporting, and five (7, 9, 10, 12, 27) (83.3%) for other biases. Only one study (11) was found at high risk of other biases because it reported inequality at baseline. The specific item scores for each study are shown in Supplementary Figure 1. The funnel plots of each outcome indicator were asymmetrical, which might be due to the small number of studies. The Egger test result are shown in Supplementary Figures 2–4.

**Figure 3:**
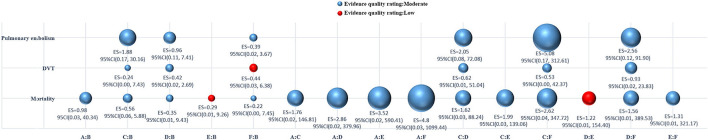
Bubble plots of the quality of evidence assessment for all comparisons. DVT, deep vein thrombosis; ES, effect size; CI, confidence interval; A, erythropoietin (12,000u); B, placebo; C, erythropoietin (30,000u); D, erythropoietin (40,000u); E, erythropoietin (70,000u); F, erythropoietin (80,000u).

Figure 3 shows the GRADE rating of the quality of evidence. The overall quality of evidence for mortality, DVT, and pulmonary embolism was moderate in both EPO and placebo groups. Some of the individual comparison groups in mortality and DVT groups were judged to have a low quality of evidence.

### Statistical analysis

**Figure 4:**
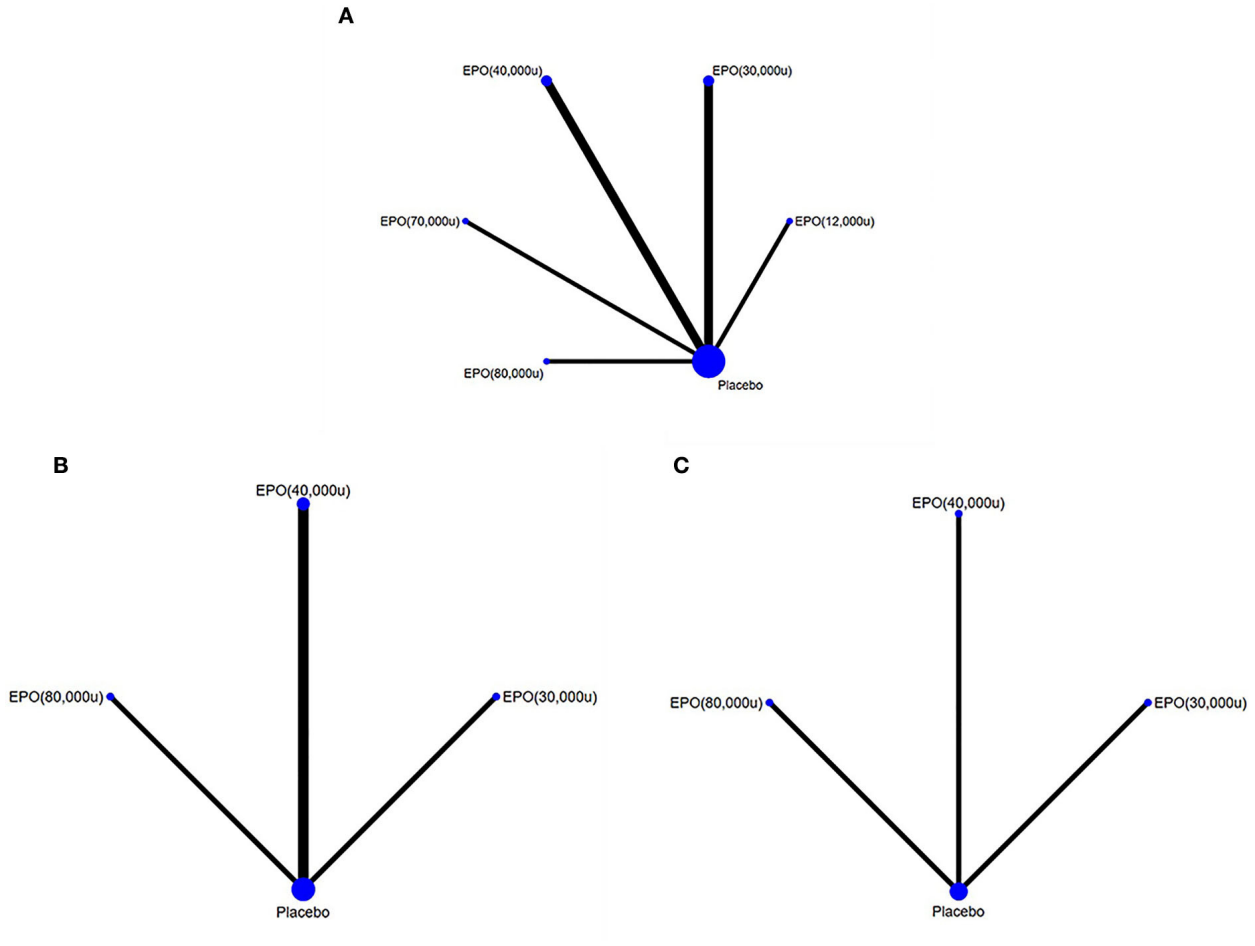
Network comparison plots for outcome indicators. **(A)** mortality, **(B)** deep vein thrombosis, and **(C)** pulmonary embolism. EPO, erythropoietin; u, unit.

**Figure 5:**
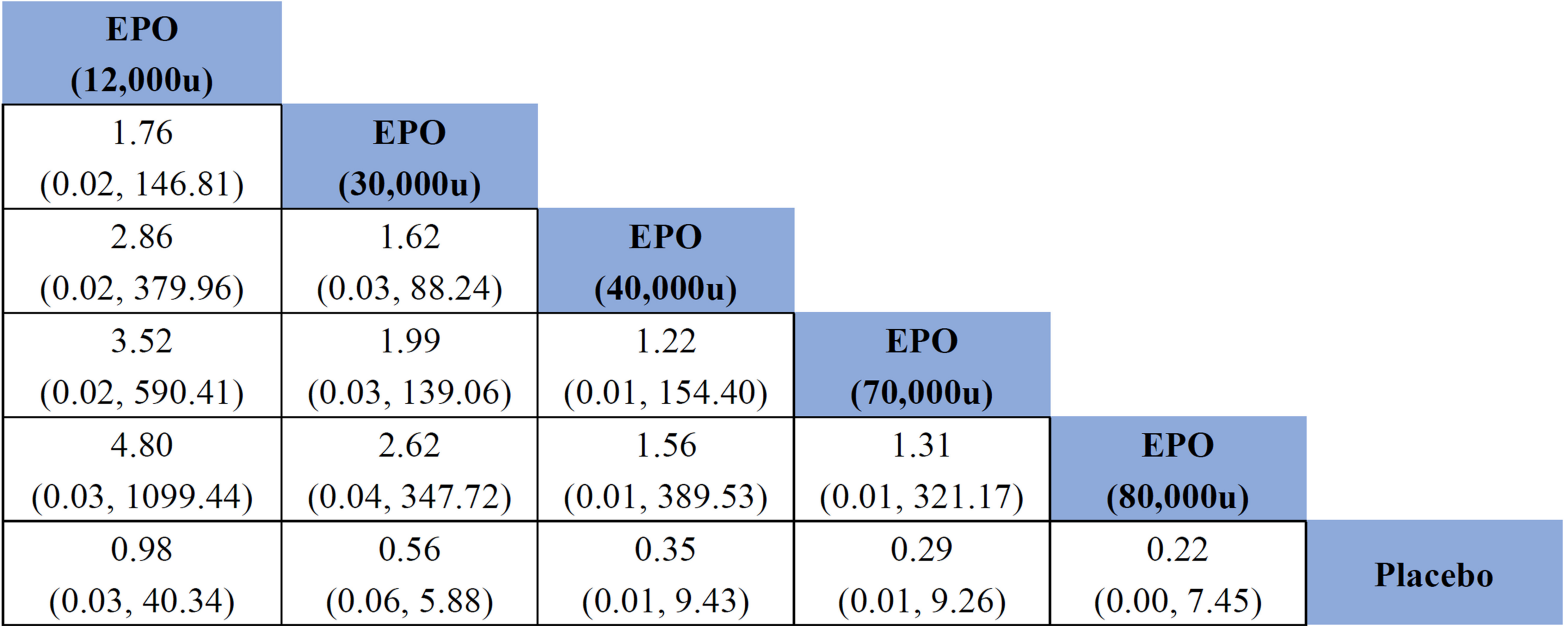
Effects of different doses of erythropoietin on mortality compared with placebo based on network meta-analysis. EPO, erythropoietin; u, unit.

Comparisons between all doses of EPO and placebo are presented in a network plot in Figure 4. Placebo served as a mediator to establish comparisons between all doses. Pooled ORs and 95% CIs of the treatment efficacy across groups in the network meta-analysis are given in Figure 5 and Supplementary Figure 5. The following specific indicators were analyzed.

#### Mortality

All studies (981 patients) reported outcomes related to mortality. The consistency analysis showed almost no statistical difference, and it was not necessary to perform the node splitting method to test the inconsistency of node branches. EPO did not significantly reduce mortality in patients with TBI compared with placebo (OR of EPO (12,000u) vs. placebo = 0.98, 95% CI: 0.03–40.34; OR of EPO (30,000u) vs. placebo = 0.56, 95% CI: 0.06–5.88; OR of EPO (40,000u) vs. placebo = 0.35, 95% CI: 0.01–9.43; OR of EPO (70,000u) vs. placebo = 0.29, 95% CI: 0.01–9.26; OR of EPO (80,000u) vs. placebo = 0.22, 95% CI: 0.00–7.45). As seen in the generated sequence diagram (Supplementary Figure 6), EPO (80,000u) had the highest therapeutic effect on patients with TBI. Figure 5 shows that the comparative effect values gradually decreased with increasing EPO dose, suggesting that the risk of death in patients with TBI tended to decrease with an increasing EPO dose.

#### Deep vein thrombosis

Only two studies (11, 27) (619 patients) reported the occurrence of DVT. The consistency analysis showed no statistical differences or closed links between node networks. EPO did not significantly increase the incidence of DVT in patients with TBI compared with placebo [OR of EPO (30,000u) vs. placebo = 0.24, 95% CI: 0.00–7.43; OR of EPO (40,000u) vs. placebo = 0.42, 95% CI: 0.02–2.69; OR of EPO (80,000u) vs. placebo = 0.44, 95% CI: 0.03–6.38]. As seen in the generated sequence diagram (Supplementary Figure 7), EPO (30,000u) posed the least risk of increased thrombotic risk in patients with TBI. Supplementary Figure 5 shows that the comparative effect values progressively decrease with increasing EPO dose, suggesting that the likelihood of deep vein thrombosis rises gradually with increasing EPO dose.

#### Pulmonary embolism

The occurrence of pulmonary embolism was reported in only one study (603 patients). The consistency analysis showed no statistical differences or closed links between node networks. EPO did not increase the risk of pulmonary embolism in patients with TBI compared with placebo (OR of EPO (30,000u) vs. placebo=1.88, 95% CI, 0.17–30.16; OR of EPO (40,000u) vs. placebo=0.96, 95% CI, 0.11–7.41; OR of EPO (80,000u) vs. placebo = 0.39, 95% CI, 0.02–3.67). As seen in the generated sequence diagram (Supplementary Figure 8), EPO (80,000u) had the highest therapeutic effect in patients with TBI. Supplementary Figure 5 shows that EPO tends to change from deleterious to beneficial with increasing doses.

## Discussion

This study compared the efficacy and safety of different doses of EPO and placebo in treating patients with TBI. EPO had a significant trend advantage in the prognosis of patients. EPO did not significantly reduce mortality in patients with TBI compared with placebo but showed a trend toward lower mortality in patients with TBI as the total dose increased. At this time, we were unable to determine the relationship between different doses of EPO and embolic events.

EPO drugs have been developed over several generations, but the original studies did not describe the specific type or batch of EPO used in detail. Each generation of EPO has a different half-life, and the variety of injection methods can affect the metabolic process of EPO in the body. However, the short half-life of EPO does not produce significantly different results among studies due to the factors mentioned earlier (28). The pharmacokinetic properties of EPO were fully considered, and our analysis collected much of the evidence on the efficacy of different doses of EPO for treating TBI, rather than the duration of treatment.

Our network meta-analysis showed that EPO did not show a significant advantage in reducing mortality in patients with TBI. Liu's research (18) found that EPO significantly reduced mid-term mortality (6 months) in patients with TBI. The main reason for the difference in conclusions may be that this study excluded RCT studies that did not accurately report drug doses and did not differentiate time to death. There were also differences in the mechanisms between the traditionally paired meta-analysis and the Bayesian algorithm-based network meta-analysis. However, robust evidence in the network meta-analysis showed that the effect value gradually decreased with the increase in the total dose of EPO, strongly implying that the higher dose of EPO was more likely to reduce the mortality of patients with TBI. Although none of the original studies have demonstrated the effectiveness of EPO treatment in patients with TBI (7, 9–12, 27), the high dose of EPO (120,000u) used in Nichol's study (27) demonstrated the narrowest confidence interval with the highest level of evidence (16), which was similar to the results of our network meta-analysis. We believe that further dose trials are warranted. Nevertheless, we were not able to detect the exact size differences between the various dosing regiments even with such a network meta-analysis.

Concerning the occurrence of deep vein thrombosis, our research showed that EPO use did not increase the risk of its occurrence, which was similar to the results of previous conventional meta-analyses (15–18). With further studies or increased doses, EPO may become a risk factor for the increased incidence of DVT in patients with TBI. Due to the small number of studies and the insignificant relationship, we believe that EPO did not increase the likelihood of pulmonary embolism, which is similar to the results of previous traditional meta-analyses (15). Notably, EPO was associated with increased blood viscosity, elevated hemoglobin concentration, and vasoconstriction, which may contribute to thromboembolism and the risk of cardiovascular events, including death (8). Although this has not yet been demonstrated in patients with TBI, we found that particular propensity and inappropriate dosage may increase the risk of embolism in this high-risk group. Our study aims to determine the optimal EPO dose for patients with TBI to ensure a reduction in adverse events based on optimal therapeutic effects.

Other related indicators were reported in the original studies, such as neurological recovery, length of hospital stay, and cardiovascular events. However, due to their small amount, they were not able to constitute the comparison network. Although most animal experiments have found that EPO can improve the prognosis of neurological function after TBI treatment, in recent meta studies, the improvement of neurological function has no statistical significance in human studies, which may be related to insufficient sample size and the number of studies or the failure to adopt the most appropriate clinical observation indicators. Liu's research (18) found that EPO has a certain effect on shortening the length of hospital stay of patients. High-quality evidence is relatively scarce, and further studies are needed to address these uncertainties, especially regarding optimal dosage, therapeutic time window, and duration of therapy.

### Limitations

There are also some limitations to our studies. First and foremost, we had to abandon a few studies where complete data cannot be obtained because of the need for relatively accurate dose calculations. In addition, we neither have complete data on what treatments patients received other than EPO nor do we know whether these essential treatments potentially impact patient outcomes. Moreover, some of the studies had a high risk of bias, but after careful assessment, this had a limited impact on the results.

## Conclusion

The network meta-analysis neither showed a significant therapeutic effect of EPO in patients with TBI nor increased the risk of adverse events. However, there is a potential correlation between EPO dose and treatment efficacy, and higher doses of EPO may be more effective in treating patients with TBI. We look forward to more high-quality original studies on the dose and timing of EPO for patients with TBI to obtain more substantial evidence on the optimal dose of EPO.

## Data availability statement

The original contributions presented in the study are included in the article/Supplementary material, further inquiries can be directed to the corresponding author.

## Author contributions

QZ and DD: literature search, literature screening, risk of bias assessment, and manuscript writing. JX and XW: analysis of data, interpretation results, and editing the manuscript. YG and LX: extraction of data and assessment of articles quality. JY and SW: figure and table production. XL: designing of study, solving the problem, and reviewing the manuscript. All authors contributed to the article and approved this version of article.

## References

[1] Ladak AA Enam SA Ibrahim MT. A review of the molecular mechanisms of traumatic brain injury. World Neurosurg. (2019) 131:126–32. 10.1016/j.wneu.2019.07.039 31301445

[2] Liu SW Huang LC Chung WF Chang HK Wu JC Chen LF . Increased risk of stroke in patients of concussion: a nationwide cohort study. Int J Environ Res Public Health. (2017) 14:230. 10.3390/ijerph14030230 28245607 PMC5369066

[3] Galgano M Toshkezi G Qiu X Russell T Chin L Zhao LR. Traumatic brain injury: current treatment strategies and future endeavors. Cell Transplant. (2017) 26:1118–30. 10.1177/0963689717714102 28933211 PMC5657730

[4] Khellaf A Khan DZ Helmy A. Recent advances in traumatic brain injury. J Neurol. (2019) 266:2878–89. 10.1007/s00415-019-09541-4 31563989 PMC6803592

[5] Skrifvars MB Bailey M Moore E Mårtensson J French C Presneill J . A post hoc analysis of osmotherapy use in the erythropoietin in traumatic brain injury study-associations with acute kidney injury and mortality. Crit Care Med. (2021) 49:e394–403. 10.1097/CCM.0000000000004853 33566466 PMC7963441

[6] Kinoshita K. Traumatic brain injury: pathophysiology for neurocritical care. J Intens Care. (2016) 4:29. 10.1186/s40560-016-0138-3 27123305 PMC4847183

[7] Bai XF Gao YK. Recombinant human erythropoietin for treating severe traumatic brain injury. Medicine. (2018) 97:e9532. 10.1097/MD.0000000000009532 29505528 PMC5943123

[8] Jelkmann W. Physiology and pharmacology of erythropoietin. Transfus Med Hemother. (2013) 40:302–9. 10.1159/000356193 24273483 PMC3822280

[9] Abrishamkar S Safavi M Honarmand A. Effect of erythropoietin on Glasgow Coma Scale and Glasgow Outcome Sale in patient with diffuse axonal injury. J Res Med Sci. (2012) 17:51–6. 23248657 PMC3523438

[10] Aloizos S Evodia E Gourgiotis S Isaia EC Seretis C Baltopoulos GJ. Neuroprotective effects of erythropoietin in patients with severe closed brain injury. Turk Neurosurg. (2015) 25:552–8. 10.5137/1019-5149.JTN.9685-14.4 26242331

[11] Nirula R Diaz-Arrastia R Brasel K Weigelt JA Waxman K. Safety and efficacy of erythropoietin in traumatic brain injury patients: a pilot randomized trial. Crit Care Res Pract. (2010) 2010:209848. 10.1155/2010/209848 20948886 PMC2951080

[12] Li ZM Xiao YL Zhu JX Geng FY Guo CJ Chong ZL . Recombinant human erythropoietin improves functional recovery in patients with severe traumatic brain injury: a randomized, double blind and controlled clinical trial. Clin Neurol Neurosurg. (2016) 150:80–3. 10.1016/j.clineuro.2016.09.001 27611985

[13] Robertson CS Hannay HJ Yamal JM Gopinath S Goodman JC Tilley BC . Effect of erythropoietin and transfusion threshold on neurological recovery after traumatic brain injury: a randomized clinical trial. Jama. (2014) 312:36–47. 10.1001/jama.2014.6490 25058216 PMC4113910

[14] Lumley T. Network meta-analysis for indirect treatment comparisons. Stat Med. (2002) 21:2313–24. 10.1002/sim.1201 12210616

[15] Liu M Wang AJ Chen Y Zhao G Jiang Z Wang X . Efficacy and safety of erythropoietin for traumatic brain injury. BMC Neurol. (2020) 20:399. 10.1186/s12883-020-01958-z 33138778 PMC7604969

[16] Liu C Huang C Xie J Li H Hong M Chen X . Potential efficacy of erythropoietin on reducing the risk of mortality in patients with traumatic brain injury: a systematic review and meta-analysis. Biomed Res Int. (2020) 2020:7563868. 10.1155/2020/7563868 33178833 PMC7644316

[17] Katiyar V Chaturvedi A Sharma R Gurjar HK Goda R Singla R . Meta-analysis with trial sequential analysis on the efficacy and safety of erythropoietin in traumatic brain injury: a new paradigm. World Neurosurg. (2020) 142:465–75. 10.1016/j.wneu.2020.05.142 32450313

[18] Liu WC Wen L Xie T Wang H Gong JB Yang XF. Therapeutic effect of erythropoietin in patients with traumatic brain injury: a meta-analysis of randomized controlled trials. J Neurosurg. (2017) 127:8–15. 10.3171/2016.4.JNS152909 27367243

[19] Page MJ Moher D Bossuyt PM Boutron I Hoffmann TC Mulrow CD . PRISMA 2020 explanation and elaboration: updated guidance and exemplars for reporting systematic reviews. BMJ. (2021) 372:n160. 10.1136/bmj.n160 33781993 PMC8005925

[20] Stang A. Critical evaluation of the Newcastle-Ottawa scale for the assessment of the quality of nonrandomized studies in meta-analyses. Eur J Epidemiol. (2010) 25:603–5. 10.1007/s10654-010-9491-z 20652370

[21] Lo CK Mertz D Loeb M. Newcastle-Ottawa Scale: comparing reviewers' to authors' assessments. BMC Med Res Methodol. (2014) 14:45. 10.1186/1471-2288-14-45 24690082 PMC4021422

[22] Balshem H Helfand M Schünemann HJ Oxman AD Kunz R Brozek J . GRADE guidelines: 3. Rating the quality of evidence. J Clin Epidemiol. (2011) 64:401–6. 10.1016/j.jclinepi.2010.07.015 21208779

[23] Chaimani A Higgins JP Mavridis D Spyridonos P Salanti G. Graphical tools for network meta-analysis in STATA. PLoS ONE. (2013) 8:e76654. 10.1371/journal.pone.0076654 24098547 PMC3789683

[24] Xu C Niu Y Wu J Gu H Zhang C. Software and package applicating for network meta-analysis: a usage-based comparative study. J Evid Based Med. (2018) 11:176–83. 10.1111/jebm.12264 29266878

[25] Jansen JP Fleurence R Devine B Itzler R Barrett A Hawkins N . Interpreting indirect treatment comparisons and network meta-analysis for health-care decision making: report of the ISPOR task force on indirect treatment comparisons good research practices: part 1. Value Health. (2011) 14:417–28. 10.1016/j.jval.2011.04.002 21669366

[26] Padilha S Virtuoso S Tonin FS Borba HHL Pontarolo R. Efficacy and safety of drugs for attention deficit hyperactivity disorder in children and adolescents: a network meta-analysis. Eur Child Adolesc Psych. (2018) 27:1335–45. 10.1007/s00787-018-1125-0 29460165

[27] Nichol A French C Little L Haddad S Presneill J Arabi Y . Erythropoietin in traumatic brain injury (EPO-TBI): a double-blind randomised controlled trial. Lancet. (2015) 386:2499–506. 10.1016/S0140-6736(15)00386-4 26452709

[28] Hedayati MH Norouzian D Aminian M Teimourian S Ahangari Cohan R Sardari S . Molecular design, expression and evaluation of pasylated human recombinant erythropoietin with enhanced functional properties. Protein J. (2017) 36:36–48. 10.1007/s10930-017-9699-9 28168382

