# Supplementary material for: Comparative safety of multiple doses of erythropoietin for the treatment of traumatic brain injury: A systematic review and network meta-analysis
Source: Front Neurol. 2022 Dec 13;13:998320. doi: 10.3389/fneur.2022.998320 (PMC9793776; doi:10.3389/fneur.2022.998320)
Supplement: Supplementary file 1 [file Table_1.pdf]

Supplementary files

Contents

Supplementary Table 1 2

Supplementary Figure 1..4

Supplementary Figure 2..5

Supplementary Figure 3..5

Supplementary Figure 4..6

Supplementary Figure 5..7

Supplementary Figure 6..8

Supplementary Figure 7..8

Supplementary Figure 8..8

Supplementary Table 1 Search strategy.

PubMed:	
#1	"brain injuries, traumatic"[MeSH Terms]
#2	"Craniocerebral Trauma"[MeSH Terms] OR "head injuries, penetrating"[MeSH Terms]
#3	"cerebrovascular trauma"[MeSH Terms]
#4	"intracranial hemorrhage, traumatic"[MeSH Terms]
#5	"brain hemorrhage, traumatic"[MeSH Terms]
#6	#1 OR #2 OR #3 OR #4 OR #5
#7	traumatic brain injur*[Title/Abstract] OR "brain contusion"[Title/Abstract] OR "craniocerebral trauma"[Title/Abstract] OR "cerebrovascular trauma"[Title/Abstract] OR penetrating head injur*[Title/Abstract] OR "traumatic intracranial hemorrhage"[Title/Abstract] OR "traumatic brain hemorrhage"[Title/Abstract] OR brain trauma*[Title/Abstract] OR traumatic encephalopath*[Title/Abstract] OR "TBI"[Title/Abstract] OR "TBIs"[Title/Abstract] OR concuss*[Title/Abstract]
#8	#6 OR #7
#9	"Erythropoietin"[MeSH Terms] OR "Epoetin Alfa"[MeSH Terms]
#10	"erythropoietin"[Title/Abstract] OR "epoetin alfa"[Title/Abstract] OR "EPO"[Title/Abstract] OR "darbepoetin alfa"[Title/Abstract]
#11	#9 OR #10
#12	#8 AND #11
Cochrane library:	
#1	MeSH descriptor: [Erythropoietin] explode all trees
#2	(EPO OR erythropoietin OR Epoetin Alfa OR Darbepoetin alfa):ti,ab,kw
#3	#1 OR #2
#4	MeSH descriptor: [Brain Injuries, Traumatic] explode all trees
#5	MeSH descriptor: [Craniocerebral Trauma] explode all trees
#6	MeSH descriptor: [Cerebrovascular Trauma] explode all trees
#7	MeSH descriptor: [Intracranial Hemorrhage, Traumatic] explode all trees
#8	MeSH descriptor: [Brain Hemorrhage, Traumatic] explode all trees
#9	#4 OR #5 OR #6 OR #7 OR #8
#10	(Traumatic Brain Injur* OR Brain Contusion OR Chronic Traumatic Encephalopathy OR Craniocerebral Trauma OR Cerebrovascular Trauma OR Penetrating head Injuries OR Traumatic Intracranial Hemorrhage OR Traumatic Brain Hemorrhage OR Brain Trauma* OR Traumatic Encephalopath* OR TBI OR TBIs OR concuss*):ti,ab,kw
#11	#9 OR #10
#12	#3 AND #11
Embase:	
#1	'erythropoietin'/exp
#2	'erythropoietin':ab,ti,kw OR 'epoetin alfa':ab,ti,kw OR epo:ab,ti,kw OR 'darbepoetin alfa':ab,ti,kw
#3	#1 OR #2
#4	‘traumatic brain injury'/exp

#5	‘traumatic brain injury':ab,ti,kw OR 'traumatic brain injuries':ab,ti,kw OR 'brain contusion':ab,ti,kw OR 'chronic traumatic encephalopathy':ab,ti,kw OR 'craniocerebral trauma':ab,ti,kw OR 'cerebrovascular trauma':ab,ti,kw OR 'penetrating head injury':ab,ti,kw OR 'penetrating head injuries':ab,ti,kw OR 'traumatic intracranial hemorrhage':ab,ti,kw OR 'traumatic brain hemorrhage':ab,ti,kw OR 'brain trauma':ab,ti,kw OR 'brain traumas':ab,ti,kw OR 'traumatic encephalopathy':ab,ti,kw OR 'traumatic encephalopathies':ab,ti,kw OR tbi*:ab,ti,kw OR concuss*:ab,ti,kw
#6	#4 OR #5
#7	#3 AND #6

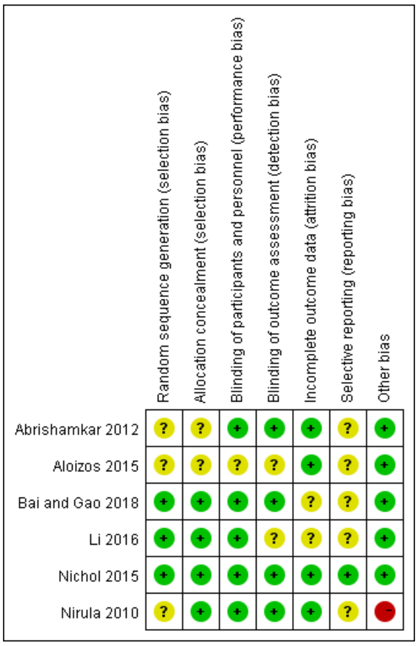

Supplementary Figure 1 Risk bias assessment of the included studies.

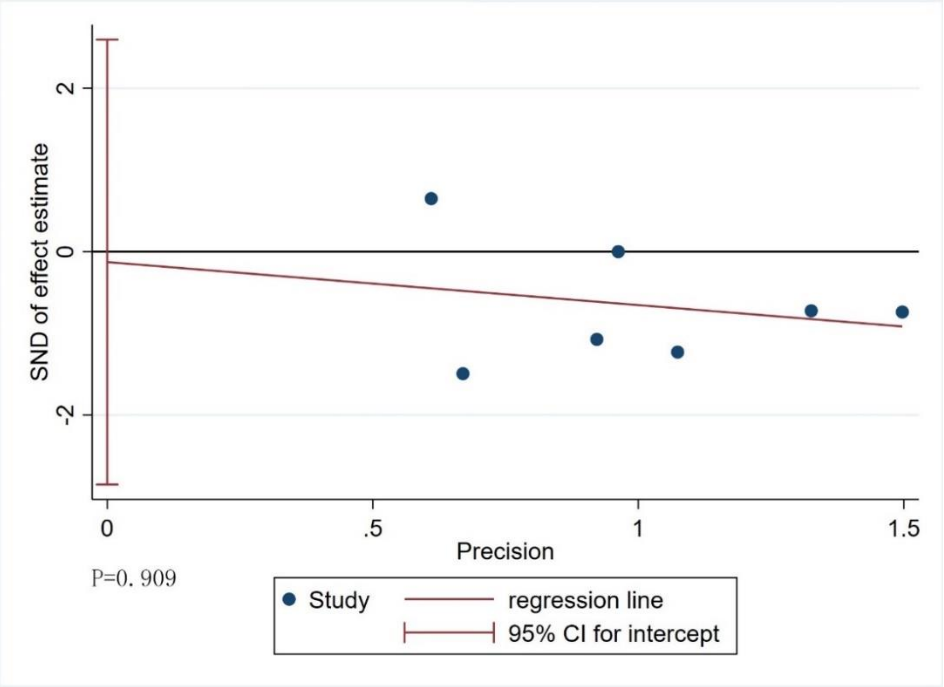

Supplementary Figure 2 Egger test for assessing publication bias in studies reporting mortality.

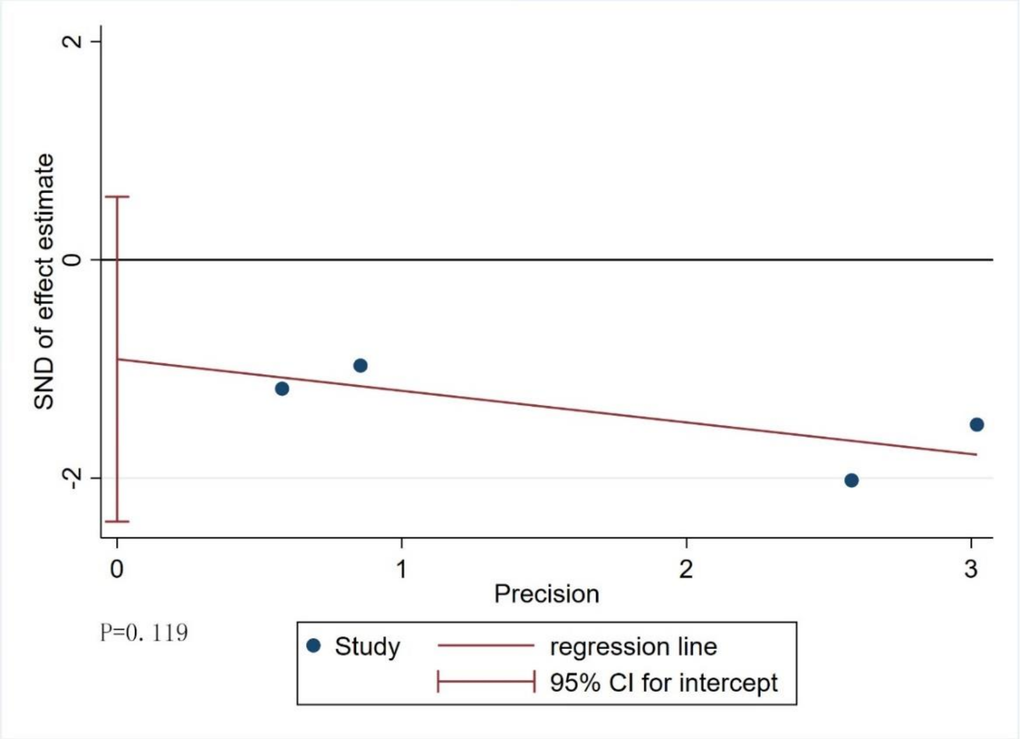

Supplementary Figure 3 Egger test for assessing publication bias in studies reporting the incidence of deep vein thrombosis.

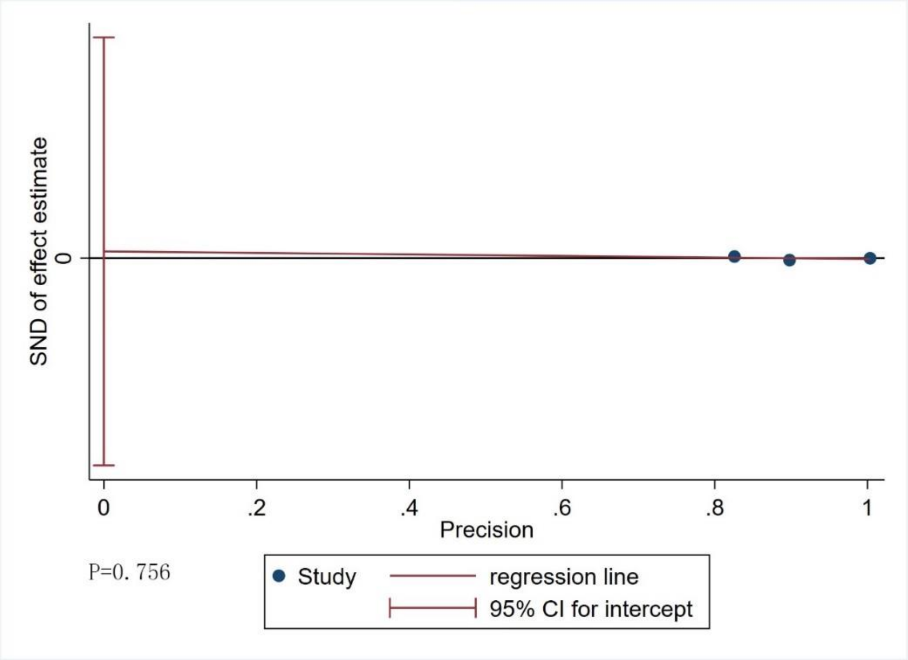

Supplementary Figure 4 Egger test for assessing publication bias in studies reporting the incidence of pulmonary embolism.

**EPO (30000u)**	2.05 (0.08, 72.08)	5.08 (0.17, 312.61)	1.88 (0.17, 30.16)
0.62 (0.01, 51.04)	**EPO (40000u)**	2.56 (0.12, 91.90)	0.96 (0.11, 7.41)
0.53 (0.00, 42.37)	0.93 (0.02, 23.83)	**EPO (80000u)**	0.39 (0.02, 3.67)
0.24 (0.00, 7.43)	0.42 (0.02, 2.69)	0.44 (0.03, 6.38)	**Placebo**

Supplementary Figure 5 Effects of different doses of erythropoietin on DVT (lower triangle) and pulmonary embolism (upper triangle) compared with placebo. *EPO* erythropoietin*; u,* unit.

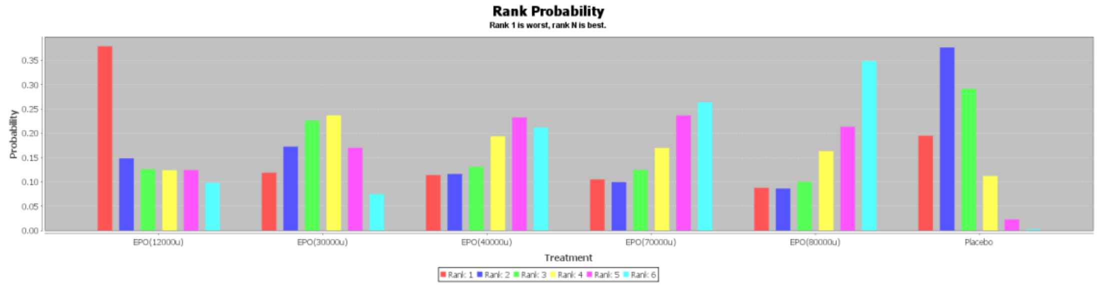

Supplementary Figure 6 Probability ranking of erythropoietin for decreasing mortality in patients with TBI. EPO, erythropoietin; u, unit; TBI, traumatic brain injury.

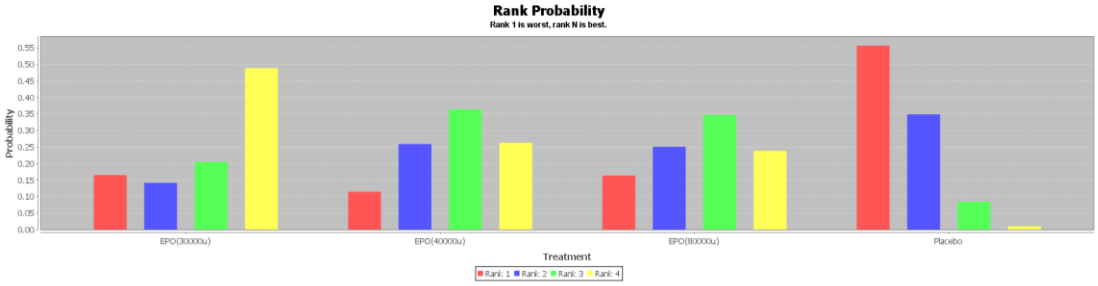

Supplementary Figure 7 Probability ranking of erythropoietin for DVT in patients with TBI. EPO, erythropoietin; DVT, deep vein thrombosis; u, unit; TBI, traumatic brain injury.

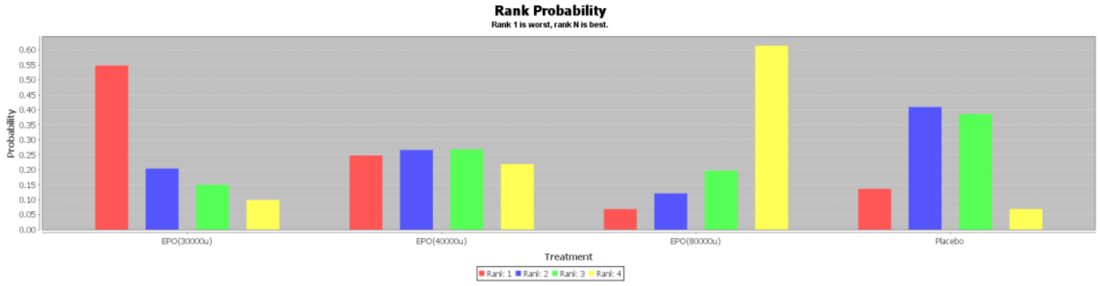

Supplementary Figure 8 Probability ranking of EPO for pulmonary embolism in patients with TBI. EPO, erythropoietin; u, unit; TBI, traumatic brain injury.
